# Trajectories of depressive symptom and its association with air pollution: evidence from the Mr. OS and Ms. OS Hong Kong cohort study

**DOI:** 10.1186/s12877-024-04731-w

**Published:** 2024-04-05

**Authors:** Yinan Zhao, Qingcai Liu, Yifei Chen, Timothy C. Y. Kwok, Jason C. S. Leung, Hui Feng, Samuel Yeung Shan Wong

**Affiliations:** 1 Xiangya School of Nursing, Central South University, Changsha, Hunan Province China; 2 Department of Medicine & Therapeutics, Faculty of Medicine, Prince of Wales Hospital, The Chinese University of Hong Kong, Hong Kong, SAR, China; 3 Xiangya-Oceanwide Health Management Research Institute, Central South University, Changsha, Hunan Province China; 4 Jockey Club Centre for Osteoporosis Care and Control, The Chinese University of Hong Kong, Hong Kong, SAR, China; 5 Jockey Club School of Public Health and Primary Care, The Chinese University of Hong Kong, Hong Kong, SAR, China

**Keywords:** Depressive symptoms, Longitudinal trajectory, Air pollutants, Older adults

## Abstract

**Background:**

Depression is a global health priority. Maintaining and delaying depressive symptoms in older adults is a key to healthy aging. This study aimed to identify depressive symptom trajectories, predictors and mortality, while also exploring the relationship between air quality and depressive symptoms in older adults in the Hong Kong community over 14 years.

**Methods:**

This study is a longitudinal study in Hong Kong. The target population was community-dwelling older adults over age 65. Depressive symptoms were measured by the Geriatric Depression Scale (GDS-15). Group-based trajectory model was used to identify heterogeneity in longitudinal changes over 14 years and examine the associations between baseline variables and trajectories for different cohort members using multinomial logistic regression. The Kaplan–Meier method was employed to conduct survival analysis and explore the variations in survival probabilities over time among different trajectory group. Linear mixed model was used to explore the relationship between air quality and depressive symptoms.

**Results:**

A total of 2828 older adults were included. Three different trajectories of depressive symptoms in older people were identified: relatively stable (15.4%), late increase (67.1%) and increase (17.5%). Female, more number of chronic diseases, poor cognitive function, and poor health-related quality of life (HRQOL) were significantly associated with other less favorable trajectories compared with participants with stable levels of depressive symptoms. The late increase group had a lower mortality rate than the relatively stable and increased groups. Lower baseline ambient air pollutant exposure to NO_2_ over 14 years was significantly associated with fewer depressive symptoms.

**Conclusions:**

In this study, we found that a late increase in depressive symptoms was the predominant trend in older Chinese people in Hong Kong. Poorer HRQOL was predictive of less favorable trajectories of depressive symptoms. Ambient air pollution was associated with depressive symptoms. This novel observation strengthens the epidemiological evidence of longitudinal changes in depressive symptoms and associations with late-life exposure to air pollution.

**Supplementary Information:**

The online version contains supplementary material available at 10.1186/s12877-024-04731-w.

## Key points

• This study was intended to capture the trajectories of depressive symptoms and the associated with air quality.

• Three different trajectories of depressive symptoms in older people were identified: relatively stable, late increase and increase trajectories.

• Female,poor cognitive function, and poorer health-related quality of life were significantly associated with other less favorable trajectories.

• After 14 years, the late increase group exhibited a lower mortality rate compared to the relatively stable and increased groups.

• Long-term exposure to NO_2_ over 14 years was significantly associated with more depressive symptoms.

**Supplementary Information:**

The online version contains supplementary material available at 10.1186/s12877-024-04731-w.

## Background

Depression is one of the highest disease burdens worldwide [1]. Globally, more than 20% of adults aged 60 and older have a mental or neurological disorder (excluding headache disorders), and 6.6% of all disabilities occurring among persons aged 60 and older are attributable to neurological and psychiatric disorders [2]. In China, the estimated loss of disability-adjusted life years (DALYs) due to depression was more than 10 million in 2013, with this figure expected to rise by 10% by 2025 [3, 4]. As the population ages, the adverse effects of depression will become more severe. A deeper understanding of the nature of depression in older populations and how to minimize it is critical to reduce this global burden. Depression is best viewed as a continuum category [5, 6]. Because the long-term trajectory of depressive symptoms varies widely across the population, some individuals have few or no symptoms, others experience transient symptoms, and others suffer from chronic depression [7]. Therefore, it is vital to determine the time point of onset and decline of depressive symptoms with strategies to delay their development and to intervene on time. Trajectory analysis of longitudinal data provides an opportunity to understand how different levels of depression appear to behave differently over time and the factors that influence them [8].

The trajectory of depressive symptoms has been used in the maternal population [9, 10], young adults [11–13], people in enforced isolation [14], recurrently depressed adults [15], and widowed elderly individuals [16]. The categorization of these trajectories varies depending on the study population and can range from 2 to 6 distinct patterns. In addition, the statistical analysis models used in each study also vary; of these, the group-based trajectory model (GBTM) is one example that allows for investigating depressive symptom trajectories [17, 18]. Despite extensive research, the trajectory of depressive symptoms among community-dwelling older adults has primarily been explored through short-term cohort studies. Therefore, more longitudinal studies on the natural courses of depression are needed, especially in older adults, where limited studies have been conducted.

Another issue is determining how different risk factors are associated with varying depression symptom patterns, which may be necessary for understanding the etiology of depression and improving treatment. Recent studies have explored the impact of a range of risk factors on depression, which can be broadly categorized into biological, psychological, and social factors. These include the number of chronic diseases [19–21], vascular factors [22, 23], health status [19, 24], disability and habits [21, 25], low self-esteem [20], low contact frequency [19], smaller network size [26], and marital status [19, 27]. With the increasing urbanization of the world’s population, there is a growing interest in studying the role of physical and social environmental factors in the development of depression [28].

In recent years, there has been a growing concern about the influence of environmental factors on depression. According to the report, air pollution caused 4.9 million deaths in 2017 and was associated with a loss of 1.47 billion Disability-Adjusted Life Years (DALYs) [29]. Indicators of poor air quality include fine particulate matter with an aerodynamic diameter of 2.5 µm or less (PM _2.5_), nitrogen dioxide (NO_2_), tropospheric ozone (O_3_), sulfur dioxide (SO_2_), and carbon monoxide (CO) [30]. The exposure to air pollutants has the potential to cause detrimental effects on the neurological system, resulting in a wide range of consequences such as alterations in behavior and the development of neurodegenerative illnesses [31, 32]. Due to widespread exposure to environmental pollutants, there is increasing evidence indicated that short-term exposure to air pollution is significantly related to residents’ depression and anxiety, was associated with increased risk of hospitalization [33–35]. However, the evidence of long-term effects of air pollution has emerged with conflicting findings [36–39]. Meanwhile, due to economic disparities, most air quality studies have been conducted in developed countries, the existence of such a relationship in developing countries is very limited [40, 41]. In addition, considering the vulnerability of older adults, they tend to have more prevalent comorbidities that may lead to broader consequences [42].

Therefore, this study aims to identify trajectories of depressive symptoms over 14 years, predictors of trajectories, and associations of different trajectories with mortality, as well as to explore the relationship between air quality and depressive symptoms among older adults in the Hong Kong community.

## Methods

### Design and study sample

All data from the Mr. OS and Ms. OS Hong Kong cohort study. The Mr. OS and Ms. OS study is the first large-scale osteoporosis study in Hong Kong and was initiated in 2001 with 2,000 men and 2,000 women [43]. After a baseline survey in 2001, the survey project conducted four waves of follow-up surveys in 2003, 2005, 2007, and 2014 [44]. From 2015 to 2017, participants were invited for a 14-year follow-up visit that included repeat questionnaire interviews and physical measurements [45]. In the current study, participants aged 65 years and older at baseline and still alive after 14 years of follow-up were included. Written informed consent was obtained from all participants, and the study was approved by the Clinical Research Ethics Committees of the Chinese University of Hong Kong.

We excluded participants who experienced mortality within 7 years of the start of the study and those who had missing data for more than 2 waves, as the limited observation time would have prevented them from developing any meaningful engagement trajectories [46]. Therefore, all participants in our analysis had at least 7 years to form trajectories before being censored. Moreover, during the process of investigating the association between air pollutants and the trajectories of depressive symptoms, participants who did not have baseline air pollutants data were excluded from the analysis. This report follows the Strengthening the Reporting of Observational Studies in Epidemiology (STROBE) reporting guidelines [47].

### Measurement

#### Depressive symptoms

Depressive symptoms were measured by the Geriatric Depression Scale (GDS-15), which is an instrument to measure depressive complaints specifically in an older population. Covinsky also used it to screen for caregiver depression [48]. The GDS-15 correlates highly with clinical depression [49, 50]. We used the short version, consisting of 15 yes or no questions. The scores were summed and ranged from 0 to 15, and the participant was categorized as having depressive complaints with a score of 5 or higher [51–53]. When evaluated against diagnostic criteria of clinical depression (AGECAT and several DSM versions), the GDS-15 appeared to have 64–98% specificity and 69–92% sensitivity [54].

#### Air pollution concentration monitoring data

Long-term hourly air pollution data with high temporal resolution recorded by the Hong Kong Environmental Protection Department (HKEPD) Local Air Quality Monitoring Network (AQMN) in the last 14 years (2001 to 2014) were collected to develop land-use regression (LUR) models for different air pollutants, as described elsewhere [55]. The gaseous air pollutants CO (carbon monoxide), NO_X_ (nitrogen oxides), SO_2_ (sulfur dioxide), and particulate air pollutants PM _2.5_ [material µm with a particle aerodynamic diameter less than or equal to 2.5, also known as fine particulate matter (FSP)] and PM _10_ [particulate matter with an aerodynamic diameter less than or equal to 10 µm, also known as respirable particulate matter (RSP)] were collected from 15 air quality monitoring stations (AQMS) of AQMN.

#### Covariates

Age, gender, chronic illness, cognitive impairment, functional impairment, social support, personality traits, and a history of depression were shown to be predictors of clinically relevant depressive symptoms [56, 57]. Based on this evidence, we selected the following covariates available in the dataset: age, sex, educational attainment, marital status, subjective social status, quality of life, cognitive function and physical function. The physical function includes body mass index (BMI), self-reported physical activity, self-reported health, fall history within the past 12 months, health behaviors and cognitive function. Additional information regarding the measures of the covariate can be found in Additional file 5.

### Statistical analyses

The sociodemographic and clinical characteristics of the sample are presented using means and standard deviations for continuous variables and frequencies for categorical variables. Participants’ depressive symptom trajectories were modeled using a group-based trajectory model (GBTM). Trajectories were derived by modeling depressive symptoms as a function of change over time. The GBTM was used to identify potential clusters of similar trajectories by analyzing the dynamics of seven-wave data, and the best-fit model was based on a Bayesian information criterion and the presence of at least 5% of participants for each trajectory to ensure stable estimation of each trajectory [17, 18]. The posterior probability of assigning each participant to a trajectory group was greater than 0.9, indicating a good model fit. A Wald test was performed to determine if the slopes of the trajectories were significantly different. The STATA Traj plug-in (Stata Corp., College Station, TX) was used to model the physical trajectories with a censored normal distribution [58].

To determine sociodemographic, clinical and the air quality predictive factors of the different trajectories of depressive symptoms, we used the LASSO algorithm (LASSO) based on the available data at baseline. A criterion of *p* < 0.05 was used to select the relevant variables to include in a multivariate model. A stepwise multinomial logistic regression model was performed to solve for the membership of the depression trajectory according to the selected relevant factors as well as baseline air quality. Additionally, The Kaplan-Meier method was employed to conduct survival analysis and explore the variations in survival probabilities over time among different trajectory group.

The third step of the analysis aimed to determine whether the air quality index, such as PM _10_ and NO_2_, were associated with depressive symptoms scores during follow-up. A linear mixed model was realized with the concentration of PM 10_10_ and the concentration of NO_2_ evaluated at each visit as the dependent variable and the depressive symptom scores as the primary independent variable. The more details showed in Additional file 6.

Additionally, we conducted a sensitivity analysis to enhance the robustness of our research findings. Initially, in order to mitigate any potential impacts of multiple imputation on the observed outcomes, participants with missing depressive symptoms data were excluded. Subsequently, a comprehensive reanalysis was performed on the resultant trajectories. Recognizing the pronounced causal relationship between disease conditions and depressive symptoms, participants with a history of pre-existing chronic conditions at baseline were subsequently excluded, including but not limited to cardiovascular diseases, stroke, and cancer.

All statistical analyses regarding associations between class membership and other variables were also performed using R statistical software, version 4.0.0 (R Project for Statistical Computing).

## Results

### Sample characteristics

**Fig. 1:**
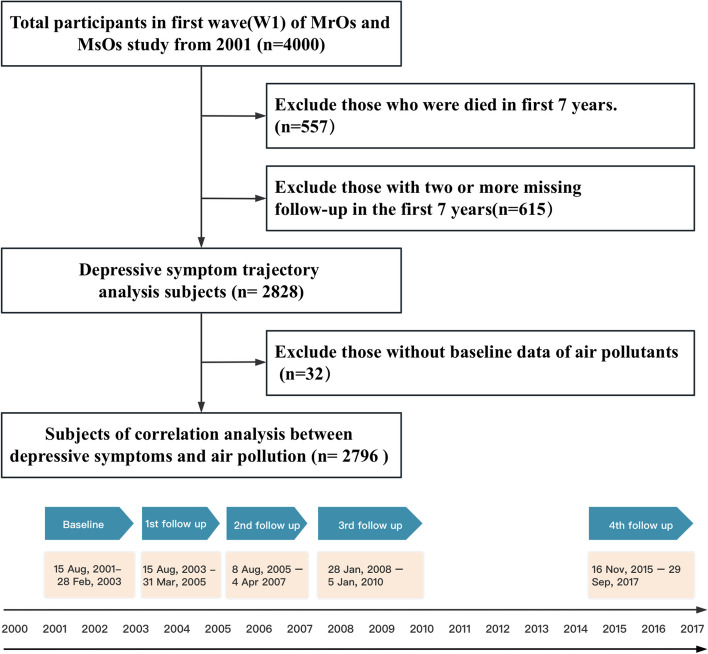
Flowchart of study population and demonstration of study timeline

**Fig. 2:**
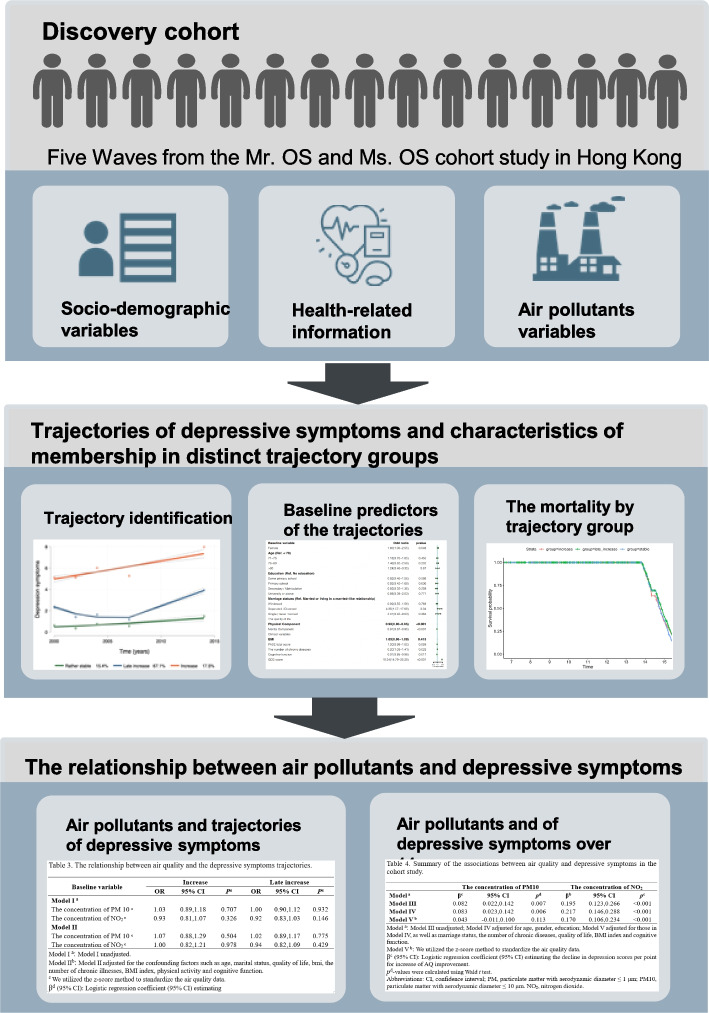
Overview of the study design

According to the selection criteria, a total of 2828 samples were included in the study (Fig. 1). Overview of the study design showed in Fig. 2. Table 1 shows the baseline characteristics of the total sample and the sample by the different trajectory groups. Compared with the excluded participants, the included people were younger, more female, higher educational level, better quality of life in the physical component, lower quality of life in mental component, higher BMI index, fewer smokers, less depressive symptoms and better cognitive function (Additional file 1). At baseline, the ages of the participants varied from 65 to 86, with a mean age of 71.65 years and a standard deviation of 4.72. The majority of participants in the study were female, with a significant proportion (69.5%) having attained a high school degree or below. A significant proportion of the participants were in a marital relationship. Initially, the median exposure to PM10 was recorded at 52.00 μg/m3, while the median exposure to NO_2_ was measured at 62.95 μg/m3.

**Table 1:** Baseline characteristics of the total sample and the sample by the different trajectory groups

Variable	Total	Groups		
		Increase (*N* = 414)	Late increase (*N* = 2023)	Relatively stable (*N* = 391)
***Socio-demographic variables***^***a***^				
Age, M (SD)	71.65 (4.72)	72.17 (4.96)	71.63 (4.70)	71.24 (4.55)
Gender, (n, %)				
Male	1380 (48.8)	141 (34.1)	1026 (50.7)	213 (54.5)
Female	1448 (51.2)	273 (65.9)	997 (49.3)	178 (45.5)
Education, (n, %)				
No education	557 (19.7)	122 (29.5)	383 (18.9)	52 (13.3)
Some primary school	913 (32.3)	132 (31.9)	681 (33.7)	100 (25.6)
Primary school	495 (17.5)	68 (16.4)	347 (17.2)	80 (20.5)
Secondary / Matriculation	558 (19.7)	63 (15.2)	399 (19.7)	96 (24.6)
University or above	305 (10.8)	29 (7.0)	213 (10.5)	63 (16.1)
Marriage statues, (n, %)				
Married or living in a married-like relationship	2063 (72.9)	269 (65.0)	1489 (73.6)	305 (78.0)
Windowed	646 (22.8)	121 (29.2)	447 (22.1)	78 (19.9)
Separated	44 (1.6)	7 (1.7)	35 (1.7)	2 (0.5)
Divorced	25 (0.9)	6 (1.4)	17 (0.8)	2 (0.5)
Single, never married	50 (1.8)	11 (2.7)	35 (1.7)	4 (1.0)
The quality of life				
Physical Component, M (SD)	49.19 (8.05)	45.91 (9.33)	49.22 (7.85)	52.48 (5.98)
Mental Component, M (SD)	56.09 (6.48)	51.51 (8.86)	56.67 (5.84)	57.99 (4.15)
***Health-related information***				
BMI, M (SD)	23.79 (3.18)	23.93 (3.25)	23.79 (3.22)	23.65 (2.91)
Self-reported health status, (n, %)				
Poor/ very poor/ fair	2662 (94.1)	357 (86.2)	1920 (94.9)	385 (98.5)
Good / excellent	162 (5.7)	57 (13.8)	103 (5.1)	6 (1.5)
The number of chronic diseases, M (SD)	1.54 (1.28)	1.80 (1.33)	1.54 (1.29)	1.28 (1.07)
PASE, M (SD)	94.31 (43.45)	88.63 (39.74)	94.73 (43.89)	98.14 (44.43)
Smoke, (n, %)				
No smoke	2666 (94.3)	395 (95.4)	1897 (93.8)	374 (95.7)
Current smoker	162 (5.7)	19 (4.6)	126 (6.2)	17 (4.3)
Drink, (n, %)				
No	2446 (86.5)	382 (92.3)	1742 (86.1)	323 (82.6)
Yes	381 (13.5)	32 (7.7)	281 (13.9)	68 (17.4)
Depression symptom, M (SD)	2.47 (2.24)	5.21 (2.42)	2.34 (1.79)	0.22 (0.57)
Cognitive function, M (SD)	25.98 (3.39)	24.72 (3.92)	26.03 (3.32)	27.04 (2.64)
***Air quality variables***^***b***^				
The concentration of PM_10_, M (SD)	52.00 (2.89)	52.05 (2.91)	51.99 (2.87)	51.98 (2.94)
The concentration of NO_2_, M (SD)	62.95 (12.98)	62.93 (12.22)	62.78 (13.03)	63.83 (13.50)

*PASE* Physical Activity Scale for the Elderly

^a^*N* = 2828

^b^*N* = 2796

### The trajectories of depressive symptoms

In each trajectory, less than 5% of the proportions in the groups were excluded, which might indicate inadequately populated classes [59]. After several data processing sessions (Additional file 2), we found that among the models in Groups 1–4, the absolute BIC values of the models in Group 3 were lower than those of the other models, and the Ave PP values were high, all being greater than 0.70. Table 2 compares the models found by GBTM for trajectory classes 1 to 4.

**Table 2:** Risk factors of depressive symptoms trajectories (relative risk ratios and 95% confidence interval)

Baseline variable	Group	
	Increase	Late increase
***Socio-demographic variables***		
Female	1.60* (1.00–2.55)	0.87 (0.61–1.24)
Age (Ref. < 70)		
71–75	1.18 (0.76–1.85)	0.97 (0.69–1.36)
76–80	1.46 (0.82–2.59)	1.17 (0.75–1.81)
> 80	1.24 (0.46–3.35)	1.07 (0.48–2.38)
Education (Ref. No education)		
Some primary school	0.85 (0.46–1.56)	1.03 (0.64–1.72)
Primary school	0.85 (0.42–1.69)	0.78 (0.47–1.40)
Secondary / Matriculation	0.66 (0.32–1.36)	0.65 (0.40–1.23)
University or above	0.88 (0.39–2.03)	0.74 (0.43–1.37)
Marriage statues (Ref. Married or living in a married-like relationship)		
Windowed/ Separated	0.92 (0.55–1.56)	0.91 (0.60–1.37)
Divorced	4.35* (1.07–17.68)	4.13* (1.25–13.66)
Single, never married	2.01 (0.45–9.03)	1.68 (0.47–6.01)
The quality of life		
Physical Component	0.93*** (0.90–0.95)	0.94*** (0.92–0.96)
Mental Component	0.91*** (0.87–0.95)	0.95* (0.92–0.99)
***Health-related information***		
BMI	1.03 (0.96–1.09)	1.02 (0.97–1.07)
PASE	1.00 (0.99–1.00)	1.00 (0.99–1.00)
The number of chronic diseases	1.20* (1.03–1.41)	1.10 (0.97–1.25)
Cognitive function	0.91* (0.85–0.98)	0.94 (0.89–1.00)

*BMI* Body mass index, *GDS* Geriatric Depression Scale, *PASE* Physical activity scale for the elderly

*p < 0.05, ***p < 0.001

**Fig. 3:**
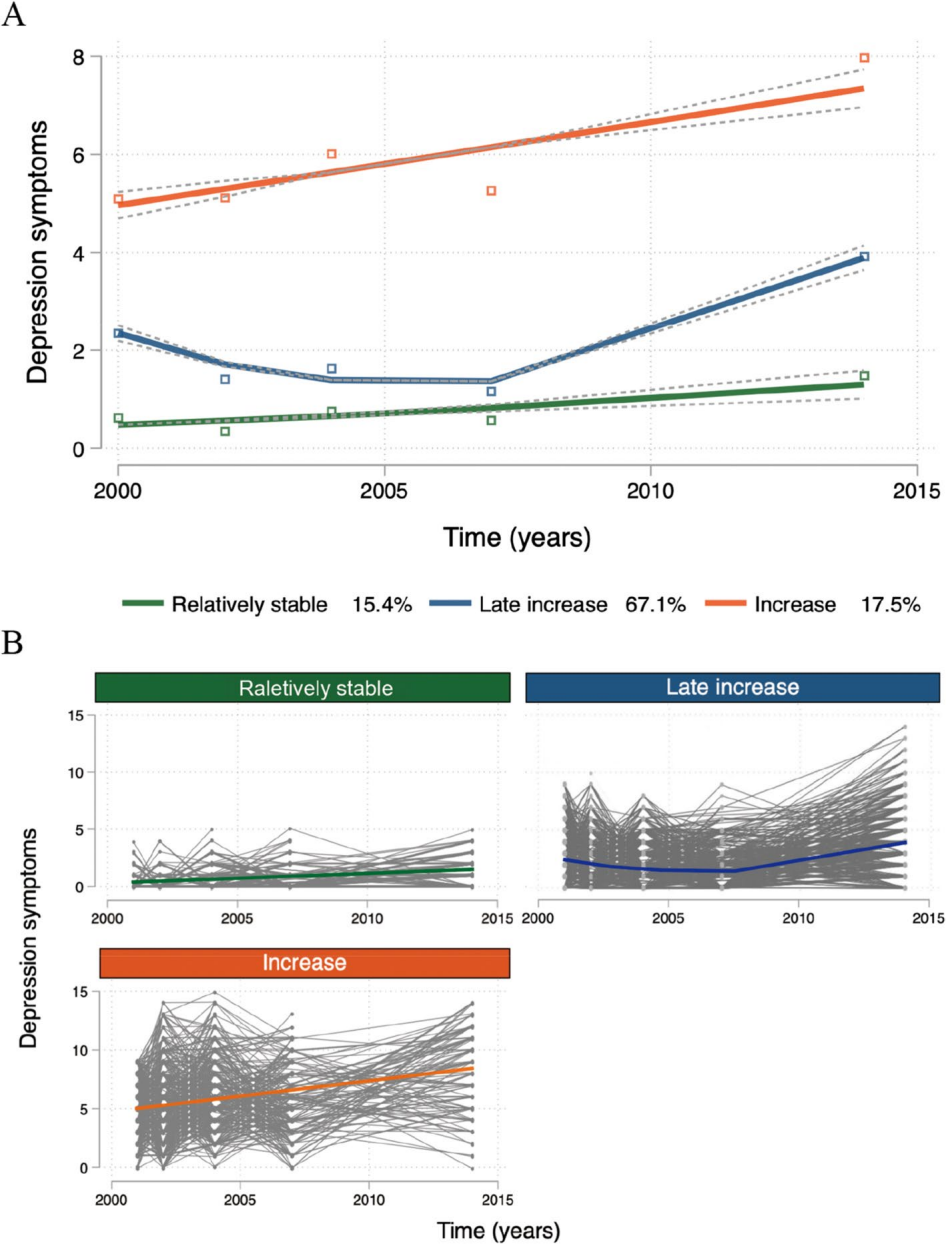
Trajectories of the depression symptom scores. (**A** Each color represents a depressive symptom trajectory. The solid lines (blue: late increase; green: raletively stable; yellow: late increase) represent estimated values. **B** Individual trajectories observed in different groups.)

Three distinct trajectories were identified over the 14-year follow-up (Fig. 3). The first trajectory consisted of 17.5% of cohort members with low depressive symptom scores at baseline who were relatively stable over time (marked as “relatively stable”). The second trajectory consisted of 15.4% of cohort members who had a slow rate of growth over 14 years (labeled “increase”). Finally, the third trajectory consisted of 67.1% of cohort members whose depressive symptom scores declined slowly in the first 7 years but increased significantly from years 7 to 14 (labeled “late increase”).

### Baseline predictors of the trajectories

**Fig. 4:**
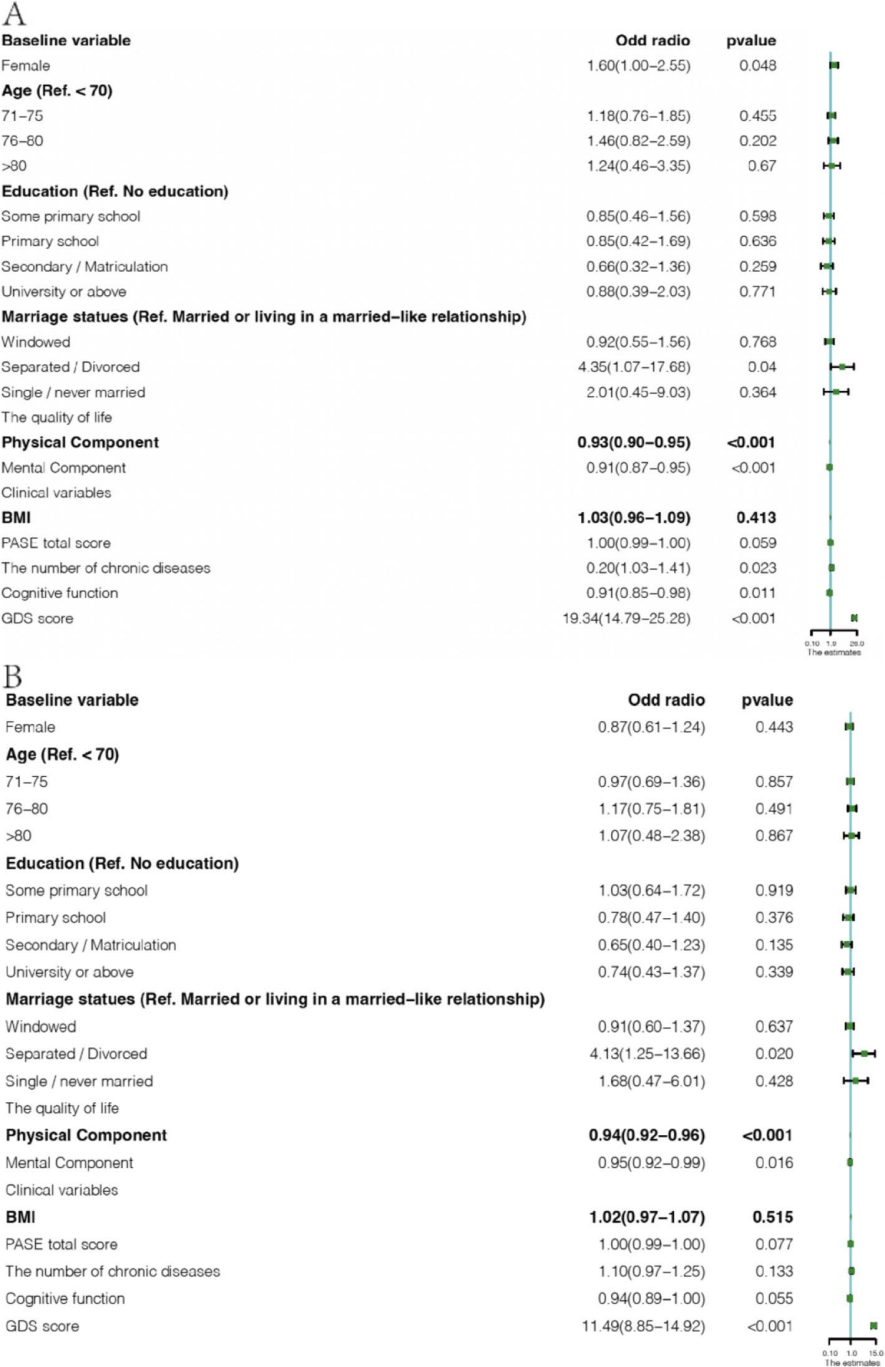
Forest plot of risk factors for depressive symptom trajectories (relative risk ratios and 95% confidence intervals). **A** group1 (stable vs increase); **B** group2 (stable vs late increase)

To identify sociodemographic and clinical predictors of the various depressive symptom trajectories, it is essential to include the most significant subset of predictors from the dataset. The LASSO regression was used to filter variables based on the available data at baseline, and more details are shown in Additional file 3. A total of 10 variables were included in the next step of the analysis. We choose “relatively stable” as the reference category because it has a relatively smooth trend in the baseline and subsequent development compared with the other three trajectories. Full results from the multivariate analysis of risk factors with varying trajectories are presented in Fig. 4 and Table 2.

The quality of life in the physical component (increase: OR,0.93; 95% CI, 0.90–0.95; late increase: OR,0.94; 95% CI, 0.92–0.96) and in mental component (increase: OR,0.91; 95% CI, 0.87–0.95; late increase: OR,0.95; 95% CI, 0.92–0.99) and the score of depressive symptoms (increase: OR,19.37; 95% CI, 14.81–25.32; late increase: OR,11.50; 95% CI, 8.85–14.93) were associated with all trajectories. Compared to the relatively stable group, the female sex (OR, 1.61; 95% CI, 1.00–2.56) and the number of chronic diseases (OR, 0.91; 95% CI, 0.85–0.98) were associated with the increased group, but they were not associated with the increased group. Separate older adults (OR, 0.91; 95% CI, 0.85–0.98) were associated with the late increase group, but they were not associated with the increase group.

### The mortality by trajectory group

**Fig. 5:**
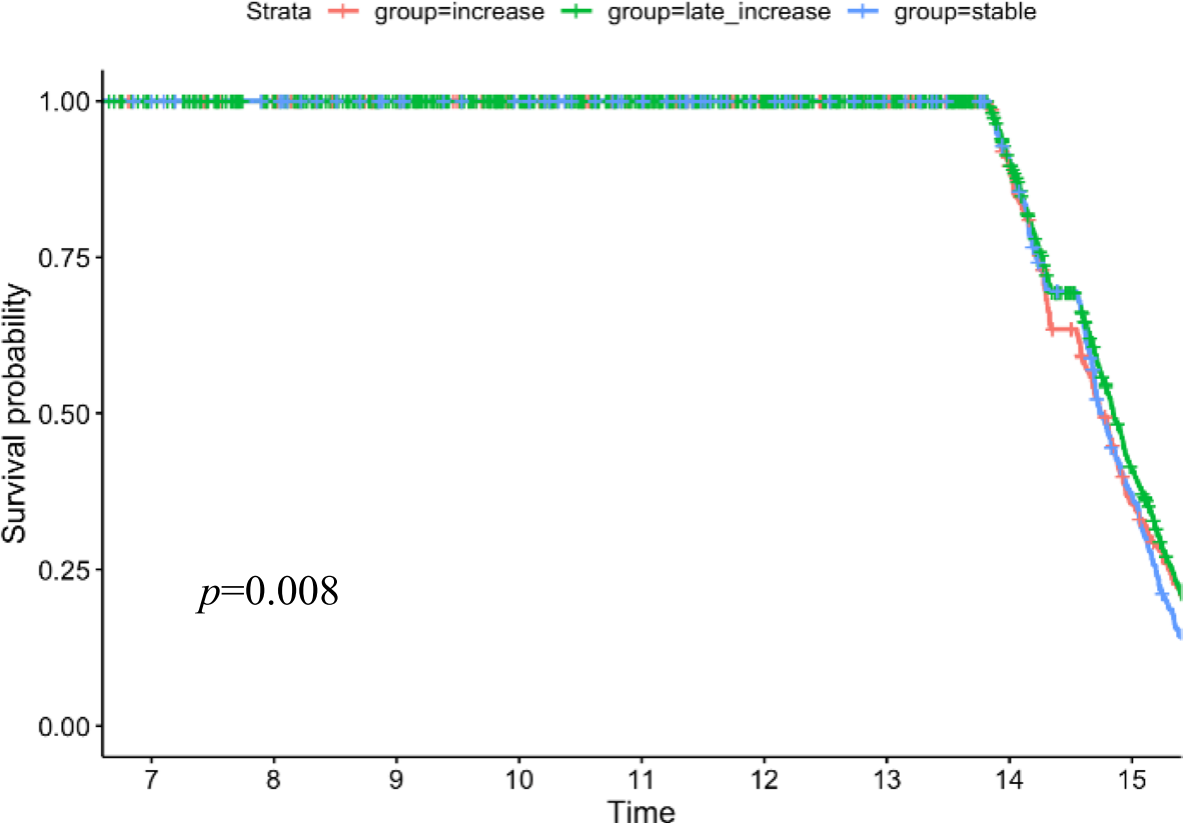
Kaplan-Meier estimate of the survival probability of mortality by trajectory group

There were markedly different risks of mortality based on trajectory group membership. We obtained stratified estimates of the cumulative incidence of mortality by trajectory group using the Kaplan‒Meier method. Time 0 is the baseline date, and patients were administratively reviewed at the time of death. The late-increase group had a lower death rate after 14 years, which was significantly different from the other two groups (*p* = 0.008) (Fig. 5).

### The relationship between air quality and depressive symptoms

The results found no significant associations between baseline ambient air pollutant exposure and either depressive symptoms at baseline or the occurrence of less favorable trajectories (Table 3). This observation remained not statistically significant after adjustment for confounding factors such as sex, marital status, quality of life (including mental and physical components), number of chronic illnesses, and cognitive function.

**Table 3:** The relationship between air quality and the depressive symptoms trajectories

**Baseline variable**	**Increase**			**Late increase**		
	**OR**	**95% CI**	***P***^***a***^	**OR**	**95% CI**	***P***^***a***^
**Model I**^a^						
The concentration of PM _10_^**c**^	1.03	0.89,1.18	0.707	1.00	0.90,1.12	0.932
The concentration of NO_2_^**c**^	0.93	0.81,1.07	0.326	0.92	0.83,1.03	0.146
**Model II**^b^						
The concentration of PM _10_^**c**^	1.07	0.88,1.29	0.504	1.02	0.89,1.17	0.775
The concentration of NO_2_^**c**^	1.00	0.82,1.21	0.978	0.94	0.82,1.09	0.429

Model I^a^: Model I unadjusted

Model II^b^: Model II adjusted for the confounding factors such as age, marital status, quality of life, bmi, the number of chronic illnesses, BMI index, physical activity and cognitive function

^c^We utilized the z-score method to standardize the air quality data

Next, we explored whether there was a correlation between depressive symptoms and air pollution over 14 years. The results of a linear mixed model exploring the link between the change in depressive symptoms and air quality. Based on fully adjusted models (Table 4, Model V), residing in locations with better ambient air quality (NO_2_: β = 0.170/year, 95% CI: 0.106–0.234) was associated with fewer depressive symptoms (Table 4).

**Table 4:** Summary of the associations between air quality and depressive symptoms in the cohort study

**Model**^**a**^	**The concentration of PM10**			**The concentration of NO**_**2**_		
	**β**^**c**^	**95% CI**	***p***^**d**^	**β**^**b**^	**95% CI**	***p***^**c**^
**Model III**	0.082	0.022,0.142	0.007	0.195	0.123,0.266	< 0.001
**Model IV**	0.083	0.023,0.142	0.006	0.217	0.146,0.288	< 0.001
**Model V**^**b**^	0.043	-0.011,0.100	0.113	0.170	0.106,0.234	< 0.001

*Abbreviations*: *CI* Confidence interval, *PM* Particulate matter with aerodynamic diameter ≤ 1 μm, *PM10* Particulate matter with aerodynamic diameter ≤ 10 μm, *NO*_*2*_ Nitrogen dioxide

Model^a^: Model III unadjusted; Model IV adjusted for age, gender, education; Model V adjusted for those in Model IV, as well as marriage status, the number of chronic diseases, quality of life, BMI index and cognitive function

Model V^b^: We utilized the z-score method to standardize the air quality data

β^c^ (95% CI): Logistic regression coefficient (95% CI) estimating the decline in depression scores per point for increase of AQ improvement

*P*^d^-values were calculated using Wald *t* test

### Sensitivity analysis

To avoid the influence of multiple imputations and selection bias on the results, we removed populations with missing data in any wave during follow-up (*n* = 2018). The proportions of the three groups and the appearance of the trajectories were similar to those in the full cohort (Additional file 4).

## Discussion

This was the first study to capturing depressive symptom trajectories in older Chinese people over 14 years. A late increase after several years of stability appeared to be the predominant trend. Only a small percentage of participants maintained a low level of depressive symptoms with a small increase over the years. Both physical and mental health-related quality of life (HRQOL) at baseline was most consistently and significantly associated with the less favorable trajectories. Females, those with more chronic diseases, and those with poorer cognitive function were more likely to have rising depressive symptoms over the next 14 years. The identified groups showed large differences in survival: after adjustment, the late increase group had a lower mortality rate than the relatively stable and increased groups. Furthermore, we found that ambient air NO_2_ exposure over 14 years was significantly associated with depressive symptoms after adjusting for several confounding variables.

Previous studies have identified 3 to 5 different trajectory categories for trajectory patterns, with approximately 10% of individuals falling into the persistent high-risk group [7, 60–62]. These variations may be due to differences in study populations, sample sizes, mythologies for identifying trajectories, and evaluation schedules [7, 60, 61, 63–70]. Two significant findings differed from previous trajectories. First, the predominant trend in the development of depressive symptoms over the 14 years was to increase in the later years, some of them are directly rising, and some of them are showing a trend of decreasing first and then rising. Only a few people maintain a low level of depressive symptoms. This highlights the increasing propensity for depression with age. This may be due to retirement, physical and cognitive decline, bereavement, reduced social networks and loss of social roles [71, 72]. Furthermore, the study revealed that elderly individuals exhibiting symptoms of depression at baseline exhibited a significant reduction in depressive symptoms over 14 years. It is plausible this is due to the inclusion of individuals who survived the first 7-year period and those who demonstrated substantial improvement in depressive symptoms and, consequently, were more likely to participate in the follow-up assessments. This finding also suggests that individuals with higher may possess better-coping mechanisms to manage the aftermath of depression, leading to an enhanced probability of reducing their depressive symptoms [73].

Understanding the underlying trajectory of health and the driver of health is critical to guiding long-term investments and policy implementation [74]. The significant predictors of depressive trajectories identified in our study were generally consistent with those in previously cited studies, such as gender and cognitive function [75, 76] Our study also revealed a robust association between quality of life and the manifestation of depressive symptoms. Previous cross-sectional studies have identified a significant association between depression and quality of life in older adults [77]. This study also adds to the evidence from longitudinal studies that found that older people with lower quality of life at baseline had a subsequent worsening of depressive conditions, even among those without previous depressive conditions. This underscores the importance of not only addressing the needs of older individuals with preexisting disease conditions but also promoting the well-being of those who are healthy. However, our study also observed similar findings in the decline group, which may be because these older individuals were already exhibiting symptoms of depression at baseline and had experienced a decline in their quality of life.

The WHO estimates that globally, ambient air pollution caused 4.2 million deaths in 2016 [78]. Several previous epidemiological studies have investigated the association between short-term exposure to ambient air pollution and the risk of depression [32, 34, 79–81]; two used inpatient data [79, 81]; four used community setting data [37, 82–84], and only one used community-dwelling older adults [37]. A cross-sectional study in China found higher levels of PM_2.5_, SO_2_ and TSP emission intensity were consistently associated with higher prevalence of depression [85]. Altug enrolled 821 older women from the German SALIA cohort and found that an increase of one interquartile range in PM_10_, PM_2.5_, NO_2_ and NO_x_ was associated with depressive symptoms assessed with the CESD-R score [86]. This article adds evidence that long-term exposure of NO_2_ is significantly associated with depressive symptom decline in elderly individuals after adjusting for several confounding variables, such as age, gender, and education level over a 14-year period. This is similar to findings from a study focused on older adults in the United States [87]. Consensus has not been reached on the pathophysiological mechanisms underlying the association between NO_2_ and depression; however, numerous paths may show that inhaling air pollutants causes oxidative stress and systemic inflammation and induces dopaminergic neurotoxicity, which leads to depressive symptoms [88–90].

The strengths of our study include the following. First, the large sample size provided by Hong Kong allowed sufficient power to identify trajectories and look for differences between trajectories. Second, we used GBTM, which can identify clusters of individuals who follow similar developmental trajectories on a given outcome by fitting a semiparametric mixture model to longitudinal data to maximize the data quality. Third, to find baseline characteristics strongly correlated with trajectory group membership, high-dimensional holdout data are incorporated into the model using machine learning algorithms. This modern robust statistical technique minimizes multicollinearity between variables. Finally, our study explored the correlation between air pollution and depressive symptoms over time-based on temporal factors.

The results of this study should be interpreted with some caution. First, the variables we used were derived from self-report surveys, which may lead to bias. However, self-reported data are commonly used in depressive symptom studies of older adults, which can accurately reflect the status of individuals interacting with the real world. Second, the ambient air pollution data in this study are based on home addresses at baseline. Participants may have moved houses, and indoor air pollution is influenced by other factors, such as building characteristics, occupant behaviors and levels of outdoor air pollution [91–93]. In addition, the decline in depressive symptoms is a long-term process. Although the 14-year cohort has been considered current, the length of the study for depressive symptom analysis is relatively long, which may result in an underestimation of the number of trajectories.

## Conclusion

The decline in depressive symptoms is a multifactorial process that includes sociodemographic characteristics, psychosocial factors, lifestyle, and indoor environment. Overall, our study shows that three trajectories of depressive symptoms have been identified in a 14-year follow-up sample of community-dwelling older adults in Hong Kong, with significant associations between gender, quality of life and cognitive function, and that increased ambient air pollution (NO_2_) is associated with the risk of an increase in depressive symptoms in community-dwelling older adults. Many researchers are paying attention to the early prevention and timely intervention of aging. These results highlight the importance of capturing these highly dimensional and heterogeneous patterns of depressive symptom development and using this information to guide depressive symptom treatment planning to develop more robust strategies to reduce the incidence of depression. This better understanding of depressive symptoms in late-life older adults could ultimately be used to effectively and efficiently tailor interventions to different groups. Targeted strategies such as more stringent air pollution guidelines and air quality control measures may help promote public mental health. This research could help maintain or slow the rate of decline in mental health and improve healthy aging.

### Supplementary Information

**Additional file 1.** Baseline characteristics of the included and excluded participants.

**Additional file 2.** The GBTM fit parameter estimates (*N* = 2828).

**Additional file 3.** Details of the process of convergence path using Lasso regression.

**Additional file 4.** Details of sensitivity analyses

**Additional file 5.** Definition and Measurement Instruments for Covariates.

**Additional file 6.** Details of linear mixed model analyses.

## Data Availability

The datasets used or analyzed during the current study are available from the corresponding author on reasonable request.
