# Supplementary material for: Trajectories of depressive symptom and its association with air pollution: evidence from the Mr. OS and Ms. OS Hong Kong cohort study
Source: BMC Geriatr. 2024 Apr 5;24:318. doi: 10.1186/s12877-024-04731-w (PMC10996234; doi:10.1186/s12877-024-04731-w)
Supplement: Supplementary file 1 — Additional file 1. Baseline characteristics of the included and excluded participants. [file 12877_2024_4731_MOESM1_ESM.docx]

Additional file 1. Baseline characteristics of the included and excluded participants.

Table S1. Baseline characteristics of the included and excluded participants.

| Variable | Entire sample  (N=4000) | Inclusion group  (N=2828) | Exclusion group  (N=1172) | *p^a^* | *p^b^* |
| --- | --- | --- | --- | --- | --- |
| ***Socio-demographic variables^a^*** |  |  |  |  |  |
| Age, M (SD) | 72.48(5.18) | 71.65(4.72) | 74.49(5.68) | <0.001 | <0.001 |
| Gender, (n, %) |  |  |  | <0.001 | 0.018 |
| Male | 2000(50.0) | 1380 (48.8) | 620(52.9) |  |  |
| Female | 2000(50.0) | 1448(51.2) | 552(47.1) |  |  |
| Education, (n, %) |  |  |  | <0.001 | <0.001 |
| No education | 856(21.4) | 557(19.7) | 299(25.5) |  |  |
| Some primary school | 1324(33.1) | 913(32.3) | 411(35.1) |  |  |
| Primary school | 683(17.1) | 495(17.5) | 188(16.0) |  |  |
| Secondary / Matriculation | 747(18.1) | 558(19.7) | 189(16.1) |  |  |
| University or above | 390(9.8) | 305(10.8) | 85(7.3) |  |  |
| Marriage statues, (n, %) |  |  |  | 0.038 | <0.001 |
| Married or living in a married-like relationship | 2829(70.7) | 2063(72.9) | 766(65.4) |  |  |
| Windowed | 987(24.7) | 646(22.8) | 341(29.1) |  |  |
| Separated | 54(1.4) | 44(1.6) | 10(0.9) |  |  |
| Divorced | 36(0.9) | 25(0.9) | 11(0.9) |  |  |
| Single, never married | 94(2.4) | 50(1.8) | 44(3.8) |  |  |
| The quality of life, M (SD) |  |  |  |  |  |
| Physical Component | 48.56(8.43) | 49.19(8.05) | 47.06(9.11) | 0.002 | <0.001 |
| Mental Component | 55.44(7.29) | 56.09(6.48) | 53.86(8.76) | <0.001 | <0.001 |
| ***Health-related information*** |  |  |  |  |  |
| BMI, M (SD) | 23.69(3.30) | 23.79(3.18) | 23.44(3.55) | 0.207 | 0.004 |
| Self-reported health status, (n, %) |  |  |  | 0.016 | <0.001 |
| Poor/ very poor/ fair | 3704(92.6) | 2662(94.1) | 1042(88.9) |  |  |
| Good / excellent | 296(7.4) | 162(5.7) | 130(11.1) |  |  |
| The number of chronic diseases | 1.61(1.30) | 1.54(1.28) | 1.78(1.33) | 0.032 | <0.001 |
| PASE | 91.32(43.01) | 94.31(43.45) | 84.12(41.07) | 0.005 | <0.001 |
| Smoke, (n, %) |  |  |  | 0.053 | <0.001 |
| No smoke | 3725(93.1) | 2666(94.3) | 1059(90.4) |  |  |
| Current smoker | 275(6.9) | 162(5.7) | 113(9.6) |  |  |
| Drink, (n, %) |  |  |  | 0.612 | 0.207 |
| No | 3477(86.9) | 2446(86.5) | 1031(88.0) |  |  |
| Yes | 522(13.1) | 381(13.5) | 141(12.0) |  |  |
| Depression symptom, M (SD) | 25.62(3.68) | 2.47(2.24) | 4.26(3.58) | <0.001 | <0.001 |
| Cognitive function, M (SD) | 2.99(2.82) | 25.98(3.39) | 24.76(4.18) | <0.001 | <0.001 |
| ***Air quality variables^b^*** |  |  |  |  |  |
| The concentration of PM10, M (SD) | 52.02(2.84) | 52.00(2.89) | 52.08(2.71) | 0.721 | 0.371 |
| The concentration of NO_2_, M (SD) | 62.8(12.89) | 62.95(12.98) | 62.44(12.69) | 0.639 | 0.252 |

Note. *p^a^* (the difference between inclusion group and the entire sample), *p^b^* (the difference between inclusion group and the excluded group). PASE, Physical Activity Scale for the Elderly; BMI, body mass index.
