# Supplementary material for: Trajectories of depressive symptom and its association with air pollution: evidence from the Mr. OS and Ms. OS Hong Kong cohort study
Source: BMC Geriatr. 2024 Apr 5;24:318. doi: 10.1186/s12877-024-04731-w (PMC10996234; doi:10.1186/s12877-024-04731-w)
Supplement: Supplementary file 2 — Additional file 2. The GBTM fit parameter estimates (N = 2828). [file 12877_2024_4731_MOESM2_ESM.docx]

Additional file 2. The GBTM fit parameter estimates (N = 2828).

Table S2. The GBTM fit parameter estimates for 1 to 4 classes using a linear function (N = 2828).

| N classes | N of parameter | AIC^b^ | BIC^c^ | Class parameter | 1 | 2 | 3 | 4 |
| --- | --- | --- | --- | --- | --- | --- | --- | --- |
| 2 | 2 | 27515 | 27533 | N | 2294 | 534 |  |  |
|  |  |  |  | % | 80.2 | 19.8 |  |  |
|  |  |  |  | *APPA^d^* | 0.9615 | 0.8848 |  |  |
|  | 3 | 27516 | 27557 | N | 2294 | 534 |  |  |
|  |  |  |  | % | 80.2 | 19.8 |  |  |
|  |  |  |  | *APPA^d^* | 0.9614 | 0.8850 |  |  |
| 3 | 3 | 27370 | 27430 | N | 1978 | 726 | 124 |  |
|  |  |  |  | % | 27.7 | 27.5 | 4.9^e^ |  |
|  |  |  |  | *APPA^d^* | 0.9102 | 0.8043 | 0.8800 |  |
|  | 4 | 27371 | 27434 | N | 1978 | 726 | 124 |  |
|  |  |  |  | % | 27.7 | 27.5 | 4.9^e^ |  |
|  |  |  |  | *APPA^d^* | 0.9102 | 0.8043 | 0.8801 |  |
|  | 5 | 27372 | 27438 | N | 1978 | 726 | 124 |  |
|  |  |  |  | % | 27.7 | 27.5 | 4.9^e^ |  |
|  |  |  |  | *APPA^d^* | 0.9105 | 0.8033 | 0.8806 |  |
|  | 6 | 26977 | 27045 | N | 752 | 1950 | 126 |  |
|  |  |  |  | % | 28.4 | 66.7 | 4.9^e^ |  |
|  |  |  |  | *APPA^d^* | 0.8267 | 0.9149 | 0.8936 |  |
|  | 7 ^a^ | 27090 | 27162 | N | 414 | 2023 | 391 |  |
|  |  |  |  | % | 15.4 | 67.1 | 17.5 |  |
|  |  |  |  | *APPA^d^* | 0.8951 | 0.8640 | 0.7248 |  |
|  | 8 | 27771 | 27845 | N | 906 | 1589 | 333 |  |
|  |  |  |  | % | 33.3 | 54.5 | 12.6 |  |
|  |  |  |  | *APPA^d^* | 0.7493 | 0.8004 | 0.8757 |  |
| 4 | 4 | 27289 | 27369 | N | 2129 | 343 | 280 | 76 |
|  |  |  |  | % | 72.9 | 13.5 | 10.9 | 2.7 |
|  |  |  |  | *APPA^d^* | 0.9366 | 0.7870 | 0.7650 | 0.8847 |
|  | 5 | 27226 | 27309 | N | 2024 | 345 | 337 | 122 |
|  |  |  |  | % | 69.1 | 14.2 | 12.3 | 4.4 |
|  |  |  |  | *APPA^d^* | 0.9245 | 0.7771 | 0.7445 | 0.8810 |

a Preferred model.

b Akaike Information Criterion.

c Bayesian Information Criterion.

d Average posterior probability of assignment.

e Groups with a proportion of less than 5%.
