# Supplementary material for: Trajectories of depressive symptom and its association with air pollution: evidence from the Mr. OS and Ms. OS Hong Kong cohort study
Source: BMC Geriatr. 2024 Apr 5;24:318. doi: 10.1186/s12877-024-04731-w (PMC10996234; doi:10.1186/s12877-024-04731-w)

Additional file 3. Details of the process of convergence path using Lasso regression.

In the study, we aimed to identify the most useful baseline characteristics for different depressive symptom groups using a LASSO logistic regression model. We established two models, namely model 1 (increase vs stable) and model 2 (late increase vs stable). Initially, we extracted 24 variables from the database, including age, sex, education level, marriage status, smoke status, social ladder in Hongkong, social ladder in community, quality of life in mental and physical components, bmi, the number of falls in last year, the score of physical function, and the number of chronic diseases, the concentrate of PM 2.5, PM10, O3 and the cognitive function. To visualize the variable selection process, we plotted the convergence path using a diagram, which is shown in Figure S1. The diagram allowed us to select an appropriate value of the tuning parameter (λ) and to identify the most important predictors for each model.

Figure S1. The Lasso regression convergence path diagram. A and C for model 1(rather stable vs increase), B and D for model 2(late increase vs rather stable).


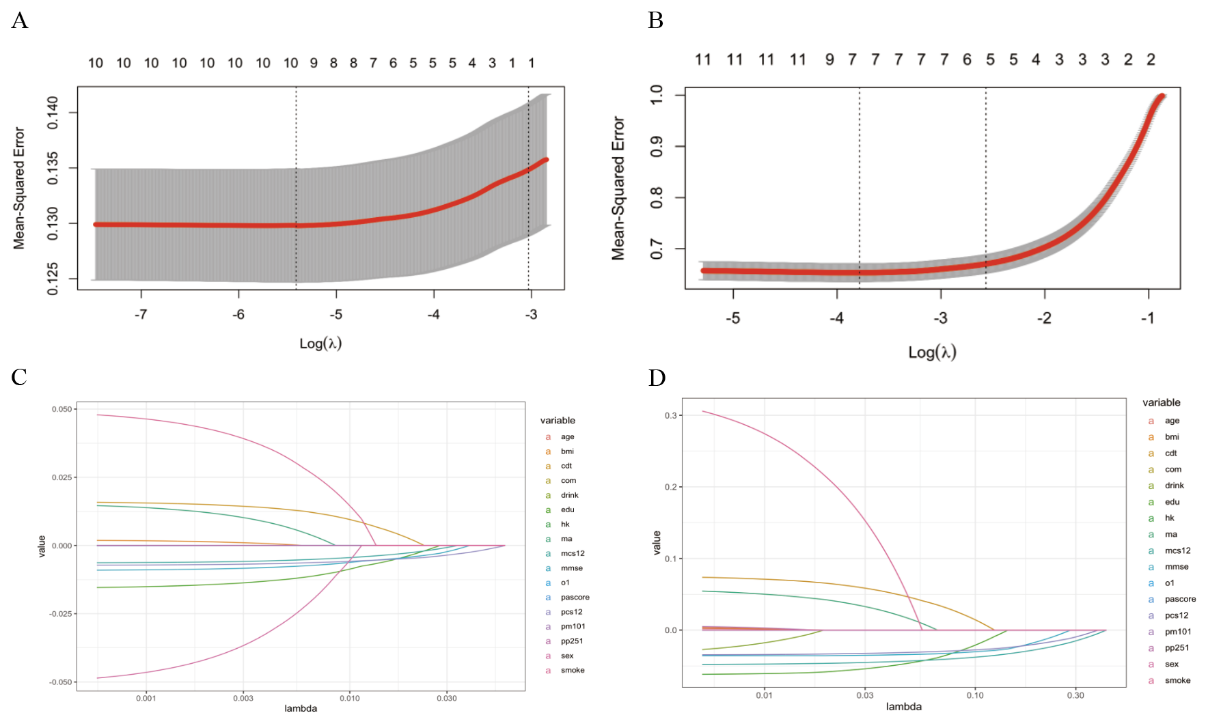

Supplement: Supplementary file 3 — Additional file 3. Details of the process of convergence path using Lasso regression. [file 12877_2024_4731_MOESM3_ESM.docx]
