# Supplementary material for: Trajectories of depressive symptom and its association with air pollution: evidence from the Mr. OS and Ms. OS Hong Kong cohort study
Source: BMC Geriatr. 2024 Apr 5;24:318. doi: 10.1186/s12877-024-04731-w (PMC10996234; doi:10.1186/s12877-024-04731-w)
Supplement: Supplementary file 4 — Additional file 4. Details of sensitivity analyses [file 12877_2024_4731_MOESM4_ESM.docx]

Additional file 4. Details of sensitivity analyses

The data imputation method is widely recognized as one of the most effective approaches for analyzing datasets with missing values, and with the increased availability of software, its popularity continues to grow [1,2]. However, there is ongoing debate about the suitability of replacing missing data with fixed values [3]. To address concerns about potential distortions from multiple imputation, we undertook an alternative approach of removing all missing values and exploring the trajectory of depressive symptoms in real data. The results, presented in Figure S2, were consistent with those obtained using multiple imputation methods.

Figure S2. The trajectory of depressive symptoms.


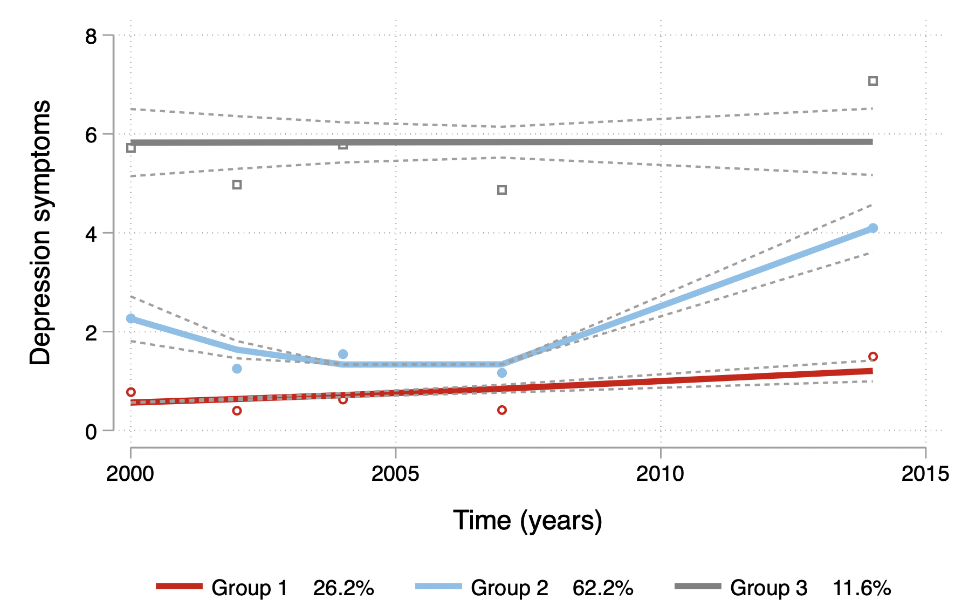


**Reference:**

1. Sterne, J. A., White, I. R., Carlin, J. B., Spratt, M., Royston, P., Kenward, M. G., ... & Carpenter, J. R. (2009). Multiple imputation for missing data in epidemiological and clinical research: potential and pitfalls. *Bmj*, *338*.

2. Graham, J. W. (2009). Missing data analysis: Making it work in the real world. *Annual review of psychology*, *60*, 549-576.

3. Baneshi, M. R., & Talei, A. R. (2012). Does the missing data imputation method affect the composition and performance of prognostic models?. *Iranian Red Crescent Medical Journal*, *14*(1), 31.
