# Supplementary material for: Trajectories of depressive symptom and its association with air pollution: evidence from the Mr. OS and Ms. OS Hong Kong cohort study
Source: BMC Geriatr. 2024 Apr 5;24:318. doi: 10.1186/s12877-024-04731-w (PMC10996234; doi:10.1186/s12877-024-04731-w)
Supplement: Supplementary file 5 — Additional file 5. Definition and Measurement Instruments for Covariates. [file 12877_2024_4731_MOESM5_ESM.docx]

Additional file 5. Definition and Measurement Instruments for Covariates.

Subjective social status was assessed using a 10-point self-anchored scale. Participants were asked to mark a picture of a standing ladder with 10 steps, with the lowest rung representing the least desirable state and the highest rung representing the most ideal state in terms of their status in the community and in Hong Kong, the ladder represents an individual's perception of their place in the community and in Hong Kong as a whole [1].

Health-related quality of life was evaluated by the 12-Item Short-Form Health Survey (SF-12), which derives summary scores from specific items from the eight domains of the short form 36, with physical component summary score (summary of physical functioning, role-physical, bodily pain, and general health) and mental component summary score (MCS) (summary of vitality, social functioning, role-emotional, and mental health) [2].

The Mini-Mental State Examination (MMSE) was used to measure cognitive function and assess cognitive impairment. The MMSE is a well-established and widely utilized instrument for evaluating cognitive abilities and detecting cognitive decline across various populations.

The MMSE comprises a set of 11 items, each designed to assess different cognitive domains, including orientation, registration, attention and calculation, recall, and language. Its scoring system ranges from 0 to 30, with lower scores indicating greater cognitive impairment [3].

**Reference:**

[1] Yu, R., Tong, C., Leung, J., & Woo, J. (2020). Socioeconomic inequalities in frailty in Hong Kong, China: a 14-year longitudinal cohort study. *International journal of environmental research and public health*, *17*(4), 1301.

[2] Lu, Z. H., Lam, F. M., Leung, J. C., & Kwok, T. C. (2022). The Resilience and the Subsequent Hospitalization of Older People with Different Frailty Status: A Prospective Cohort Study. *Journal of the American Medical Directors Association*, *23*(10), 1718-e1.

[3] Arevalo‐Rodriguez, I., Smailagic, N., i Figuls, M. R., Ciapponi, A., Sanchez‐Perez, E., Giannakou, A., ... & Cullum, S. (2015). Mini‐Mental State Examination (MMSE) for the detection of Alzheimer's disease and other dementias in people with mild cognitive impairment (MCI). *Cochrane Database of Systematic Reviews*, (3).
