# Supplementary material for: Trajectories of depressive symptom and its association with air pollution: evidence from the Mr. OS and Ms. OS Hong Kong cohort study
Source: BMC Geriatr. 2024 Apr 5;24:318. doi: 10.1186/s12877-024-04731-w (PMC10996234; doi:10.1186/s12877-024-04731-w)
Supplement: Supplementary file 6 — Additional file 6. Details of linear mixed model analyses. [file 12877_2024_4731_MOESM6_ESM.docx]

Additional file 6. Details of linear mixed model analyses

The third step of the analysis aimed to determine whether the air quality index, such as PM 10 and PM NO_2_, were associated with depressive symptoms scores during follow-up. A linear mixed model was realized with the concentration of PM10 and the concentration of NO_2_ evaluated at each visit as the dependent variable and the depressive symptom scores as the primary independent variable. The equation can be written as:

$$Y_{ij}=\alpha+\beta_{1}*\mathrm{AQ}_{\mathrm{ij}}+\beta_{2}*\mathrm{Time}_{ij}+\beta_{3}*\mathrm{AQ}_{\mathrm{ij}}*\mathrm{Time}_{ij}+\sum_{p=1}^{P} \gamma_{p}*Z_{\mathrm{ij}}+a_{ij}+\epsilon_{ij}$$

In this equation, $Y_{ij}$ represents repeated measures of depressive symptom scores, where *i* represents different time points and *j* represents individuals. The variable $\mathrm{AQ}_{\mathrm{ij}}$ represents the air quality measure for individual *j* at each time point *i*. The $\mathrm{Time}_{ij}$ variable represents the year since the enrollment for individual *j* at the *i* time point. $Z_{ij}, \ldots, Z_{ij}$ represents all other individual-level covariates included in the model. $a_{ij}\sim N(0,\varphi^{2})$ is the random intercept at the individual level. $\epsilon_{ij}\sim N(0,\sigma^{2})$ represents the error term.
